# Contribution of the gonococcal *NEIS1446-ispD* gene conversion to the pathobiology of the *Neisseria meningitidis* urethritis clade, *Nm*UC

**DOI:** 10.1128/iai.00350-24

**Published:** 2025-02-04

**Authors:** Emilio I. Rodriguez, Yih-Ling Tzeng, Soma Sannigrahi, David S. Stephens

**Affiliations:** 1 Division of Infectious Diseases, Department of Medicine, Emory University School of Medicine 234195 , Atlanta, Georgia, USA; 2 Microbiology and Molecular Genetics Program, Graduate Division of Biological and Biomedical Sciences, Emory University Laney Graduate School 310203 , Atlanta, Georgia, USA; 3 Department of Microbiology and Immunology, Emory University School of Medicine 197280 , Atlanta, Georgia, USA; Universite de Geneve, Genève, Switzerland

**Keywords:** *Neisseria meningitidis*, *Neisseria gonorrhoeae*, *Nm*UC, urogenital pathogen, gene conversion, *ispD*, isoprenoid

## Abstract

Over the last decade, a *Neisseria meningitidis* (*Nm*) urethritis-causing clade (*Nm*UC) has emerged to cause clusters of meningococcal urethritis in the United States and globally. One genomic signature of *Nm*UC is the integration of *Neisseria gonorrhoeae* (*Ng*) DNA in an operon, *NEIS1446-NEIS1438*, which partially replaced the *Nm ispD* gene. IspD is the 2-C-methyl-d-erythritol 4-phosphate cytidylyltransferase of the terpenoid precursor synthesis pathway, required for the production of ubiquinones of the electron transfer chain. IspD is essential in several gram-negative bacteria. The biological importance of the *NEIS1446-ispD* gene conversion event for *Nm*UC was investigated. The *ispD* gene was found to be essential in *Nm*UC (CNM3) and non-clade *Nm* (MC58), and a mutation at the native locus can only be made with the insertion of a second *ispD* copy in the genome. The IspD_MC58_ variant was more efficient at promoting aerobic growth at a low level than IspD_CNM3_; the two proteins differ by 15 residues. Maximal aerobic growth densities of strains with an *Nm*UC background resembled *Ng* (FA19), and both were significantly lower than *Nm*. In contrast to non-clade *Nm*, all *Nm*UC strains survived well anaerobically. Increasing *ispD* expression by titrating IPTG in non-clade *Nm* enhanced anaerobic survival. Translational reporters of the *Nm*UC and *Ng* promoters demonstrated similar expression levels, and both were significantly higher than non-clade *Nm*, under aerobic and microaerobic conditions. Our findings suggest that the integration of gonococcal DNA into the *NEIS1446-NEIS1438* operon of *Nm*UC has increased *ispD* expression*,* contributing to *Nm*UC’s adaptation to the oxygen-limited environment of the human urogenital tract.

## INTRODUCTION

*Neisseria meningitidis* (*Nm*) is an exclusively human pathogen that typically colonizes the nasopharynx. The bacterium can invade the bloodstream and cause invasive meningococcal disease (IMD), which usually manifests as meningitis, bacteremia, or sepsis (1). However, *Nm* can also colonize the rectum and urogenital tract. Outbreaks of IMD, believed to be sexually transmitted, have occurred in the last two decades in North America and Europe among populations of men who have sex with men (MSM). These outbreaks were caused by group C clonal complex 11 (cc11) isolates (2–4) and were associated with meningococcal colonization of the urethra and rectum (5, 6). Beginning in 2015, clusters of *Nm* urethritis, originally presumed to have been caused by *Neisseria gonorrhoeae* (*Ng*), were reported in multiple US cities occurring primarily in heterosexual men (7). Phylogenetic analysis of these *Nm* urethritis isolates found that they belong to the lineage 11.2 of cc11 and formed a distinct clade, designated as the *Nm* urethritis clade, *Nm*UC. The closest relatives to *Nm*UC were invasive cc11 isolates (8, 9).

Over 200 *Nm*UC isolates (2013–2016) from multiple US states were analyzed and reported in 2018 (8). Recently, additional *Nm*UC isolates have since been identified and characterized from the United States, the United Kingdom, Japan, and Vietnam (10–13). *Nm*UC has predominantly been recovered from the male urethra, but also from the rectum, pharynx, and female genital tract. Furthermore, the clade has been reported to cause neonatal conjunctivitis (14) and, rarely, invasive disease (8, 15), mostly in patients with immunocompromising conditions, including HIV infection and complement deficiency (15).

**Fig 1:**
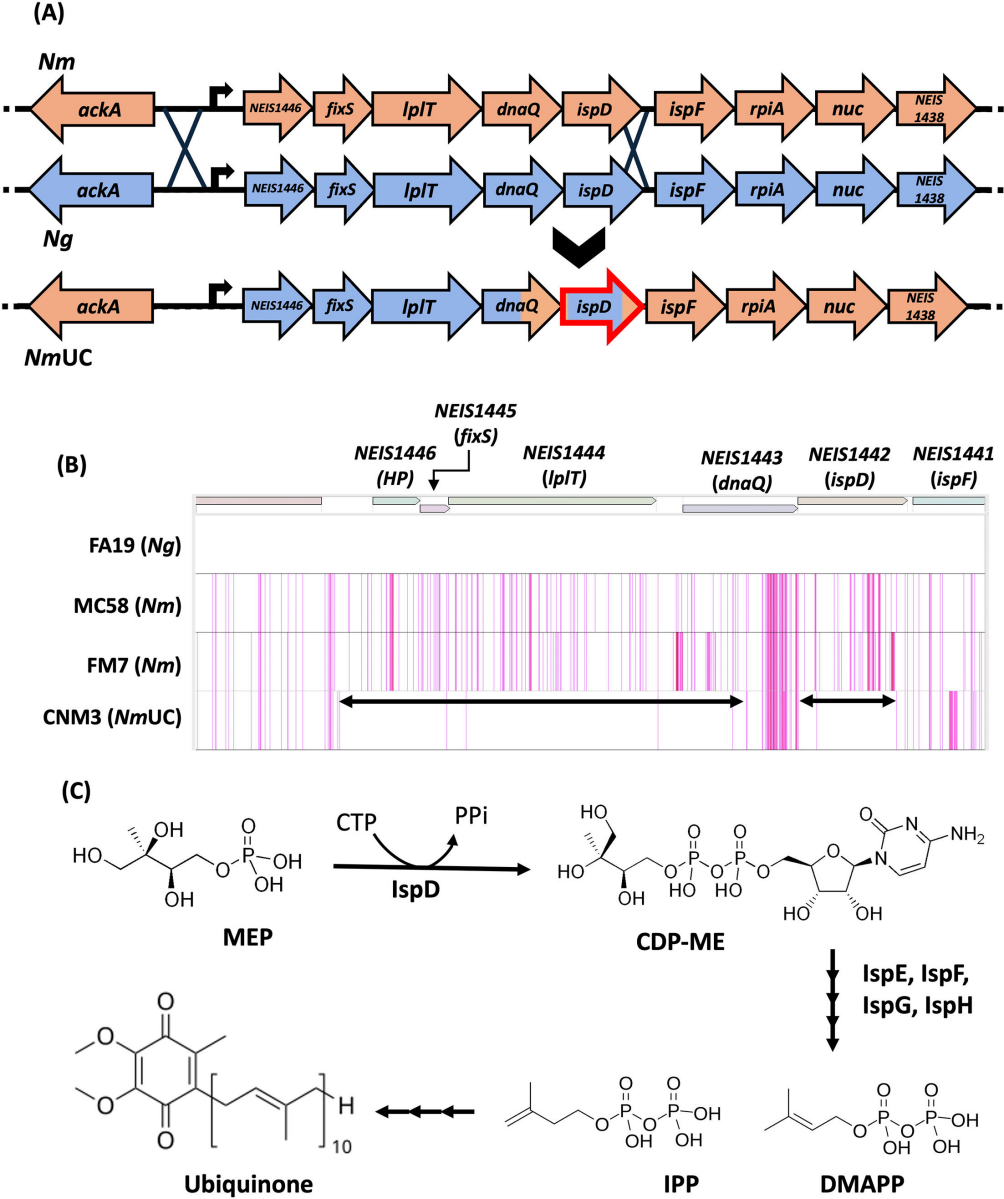
(**A**) Gene conversion in the *NEIS1446-NEIS1438* operon in *Nm*UC. The *Nm* ancestor of *Nm*UC underwent homologous recombination with *Ng* DNA, replacing five genes (*NEIS1446-ispD*) of a larger nine-gene operon and acquiring gonococcal alleles (8). Meningococcal genes are colored in orange and gonococcal genes in blue. Modified from Fig. 1B in Rodriguez et al. (11) in accordance with Creative Commons Attribution 4.0 International (CC-BY-4.0). (**B**) SNP density plots of gonococcal sequence in *Nm*UC. Core genomes were aligned with gonococcal strain FA19 set as the reference genome. Each SNP that differs from FA19 is shown as a single line, and multiple neighboring SNPs appear as thick lines. The black double-sided arrows denote the crossover sequence blocks of the recombination events with *Ng* DNA. (**C**) Production of ubiquinone via the Mevalonate-independent (MEP) pathway. IspD catalyzes the third step in the MEP pathway, converting 2-C-methylerythritol 4-phosphate (MEP) to 4-diphosphocytidyl-2-C-methylerythritol (CDP-ME). Additional enzymes in the MEP pathway, including IspF, convert CDP-ME into isoprenoid precursors: isopentenyl pyrophosphate (IPP) and dimethylallyl pyrophosphate (DMAPP). The precursors are used in the biosynthesis of isoprenoids, including ubiquinone. Modified from Fig. 1 in Xue et al. (18) in accordance with CC-BY-4.0.

Genome sequence analyses of over 250 *Nm*UC isolates have identified unique and universally shared genetic characteristics (8). An IS*1301*-associated deletion of the capsule biosynthesis genes, *cssA/B/C*, resulted in the loss of group C capsule and intrinsic sialylation of lipooligosaccharides. In addition, gonococcal DNAs had recombined into the genomes of all *Nm*UC isolates (8), resulting in several gene conversions. These gene conversion events included acquisition of the *Ng* denitrification apparatus, encoded by the divergently transcribed *aniA* and *norB*, which is important for transitioning from the aerobic nasopharyngeal environment to a microaerobic/anaerobic urethral environment (16). Denitrification provides alternative electron acceptor-driven respiration when oxygen is limited, thus facilitating microaerobic growth (17). Another universal gene conversion event identified in *Nm*UC is a five-gene fragment, *NEIS1446-ispD*, of an operon, *NEIS1446-NEIS1438* (Fig. 1A). These two genetic features are strongly maintained in the currently identified 261 *Nm*UC isolates, collected between 2013 and 2022 (11). The importance of the *NEIS1446-ispD* gene conversion event to *Nm*UC adaptation, biology, and pathogenesis is unknown and motivated us to investigate the contributions of this gene conversion to the emerging urotropic *Nm*UC.

## RESULTS

### Gene conversion events in the *NEIS1446-ispD* locus with *Ng* DNA in *Nm*UC

The ~3.3 kb gonococcal DNA fragment, previously identified as a universal conversion event (8) in *Nm*UC, is within an operon encoding proteins of diverse functions (19): *NEIS1446*, a hypothetical protein; *fixS*, a *cbb_3_*-type cytochrome oxidase maturation protein; *lplT*, the lysophospholipid transporter; *dnaQ*, the DNA polymerase III epsilon subunit; and *ispD*, the 2-C-methyl-d-erythritol 4-phosphate cytidyltransferase (Fig. 1A). Other genes*—ispF*, *rpiA*, *nuc*, and *NEIS1438*—are part of the operon but were not altered by the gene conversion event (8, 19). Upstream of *NEIS1446* and divergent in transcription is the *ack*A gene encoding an acetate kinase; when *ackA* is mutated, *Ng* is unable to grow anaerobically and has a defect in biofilm formation (20). The conversion event also changed the intergenic sequence between *ackA* and *NEIS1446* (8).

The SNP density plot showed that *NEIS1446-NEIS1442* (*ispD*) had recombined gonococcal DNA sequence(s), replacing much of the meningococcal sequence (Fig. 1B). However, *dnaQ* does have a 3′ partial meningococcal sequence (continuous stretch of 49 SNPs matching those of MC58 *dnaQ*) (Fig. 1B). This could indicate two separate recombination events of *Ng* sequence flanking *dnaQ*, or a subsequent recombination replacing the gonococcal *dnaQ* sequence with a meningococcal *dnaQ* fragment. Alternatively, a single recombination event could have occurred with a gonococcal DNA that already contained the *Nm dnaQ* sequence. However, no such a sequence was identified in the 24,762 *Ng* genomes of the PubMLST database and this *dnaQ* allele (*dnaQ*_328) is unique to the clade. Importantly, the *NEIS1446-NEIS1442* (*ispD*) recombination event(s) occurred early in the evolution of *Nm*UC as 245 out of 261 (93.9%) of *Nm*UC isolates have this configuration of *NEIS1446-NEIS1442* (11).

The *ispD* gene had 588 out of 691 nucleotides (85%) affected by the recombination, resulting in an *ispD* allele that is unique to *Nm*UC, with 99.3% nucleotide identity to the consensus *ispD* sequence of *Ng* and 93.5% nucleotide identity to *Nm*. Based on work in *Escherichia coli* (21), IspD is predicted to be a transferase that catalyzes the third step of the mevalonate-independent (MEP) pathway, responsible for the generation of isoprenoid precursors (Fig. 1C) (22). The enzyme encoded by the downstream *ispF* catalyzes the fifth step in the MEP pathway.

### The *Ng NEIS1446-ispD* genes remained co-transcribed as an operon in *Nm*UC

A previous study found that *lplT-NEIS1438* was part of a seven-gene operon in *Ng* (19). To better define the operon and see if the gene conversion event affected the presumed operon structure in *Nm*UC, we performed PCR-linkage experiments (Fig. S1). Total RNA was isolated for cDNA preparation and then conventional PCR was performed to determine if the genes were transcribed as a single mRNA transcript. Due to the length of amplification, two sets of linkage-PCR were performed to examine the nine predicted open reading frames, *NEIS1446-NEIS1438*. The data demonstrated that *NEIS1446-ispF* and *dnaQ-NEIS1438* were co-transcribed on the same transcripts. Thus, the transferred gonococcal alleles function as a 9-gene transcriptional unit in *Nm*UC (Fig. S1).

### IspD is essential for *N. meningitidis*

**Fig 2:**
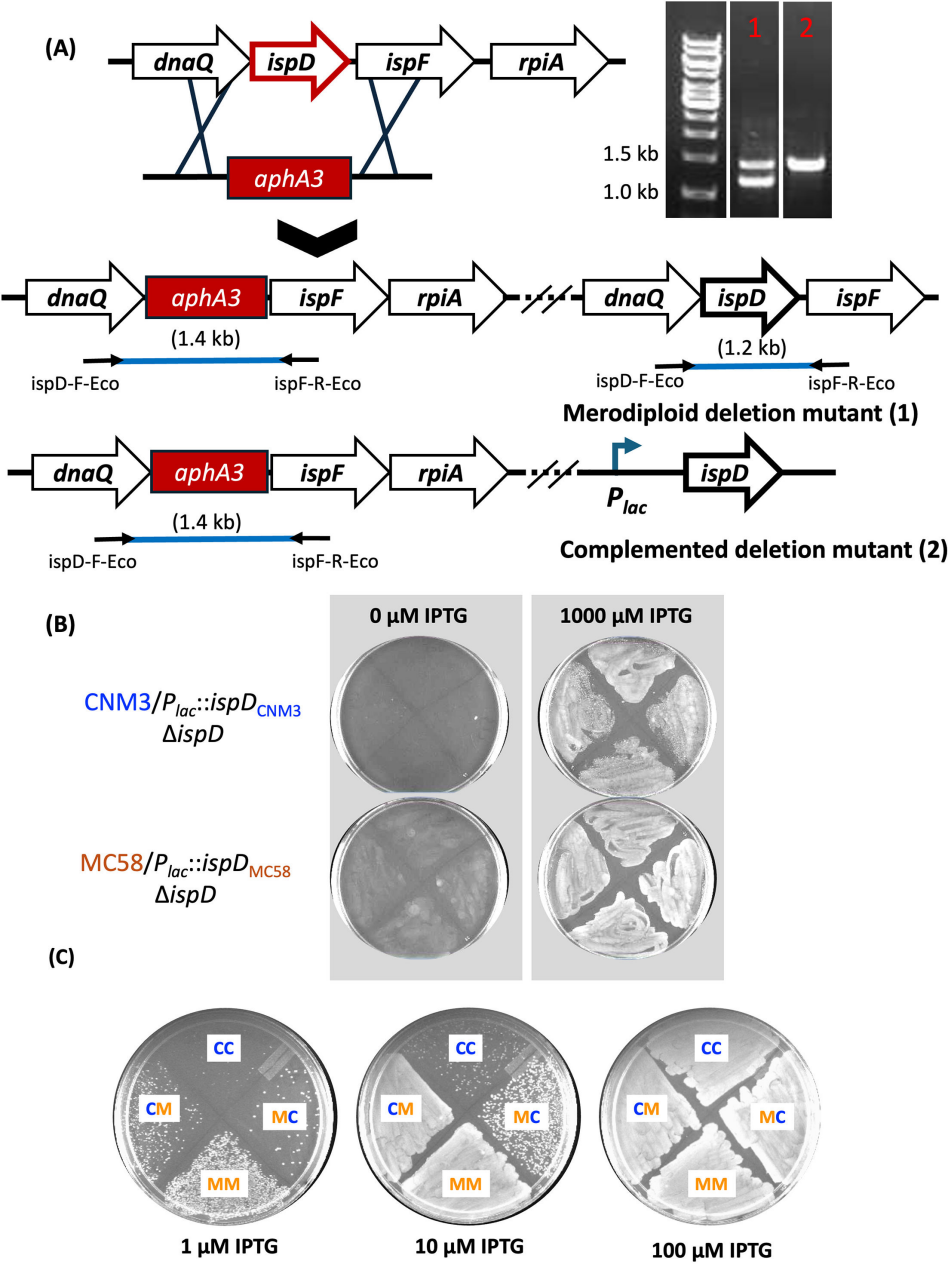
Generation of *ispD* deletion mutants and impact on aerobic growth. (**A**) Complemented *ispD* deletion mutants. Colonies with native *ispD* produce a 1.2 kb PCR product using ispD-F-Eco + ispF-R-Eco primers, while the *ispD::aphA3* configuration generates a 1.4 kb product. Deleting *ispD* in WT *Nm* selected for transformants with merodiploid for *ispD*. Inserting *ispD* under the control of an IPTG-inducible *lac* promoter at a distinct genomic location, and then deleting the native *ispD,* successfully generated a mutant with deleted native copy. Merodiploid deletion mutants have both 1.2 and 1.4 kb bands (lane 1). Complemented deletion mutants only have a 1.4 kb band (lane 2). (**B**) Complemented mutants plated on GC plates and grown aerobically with and without IPTG. Mutants were grown in broth culture to mid-log phase and spread on GC plates. Each quarter plate is a separate culture. The plates shown are representative of aerobic growth experiments repeated three times. (**C**) Complemented mutants plated on GC plates and grown aerobically with varying concentrations of IPTG. Mid-log phase cultures were spread on GC plates. The plates shown are representative of aerobic growth experiments repeated three times. CC = CNM3::*ispD_Nm_*_UC_ Δ*ispD;* CM = CNM3::*ispD_Nm_* Δ*ispD;* MM = MC58::*ispD_Nm_* Δ*ispD;* MC = MC58::*ispD_Nm_*_UC_ Δ*ispD*.

IspD has been shown to be essential in several gram-negative bacteria (23, 24). To determine if *ispD* was essential in *Nm*, a ~600-bp *ispD* coding sequence was deleted in-frame and replaced with the ~800-bp *aphA3* kanamycin resistance cassette. To assess the mutation, different PCR product lengths were expected for the wild type (WT, 1.2 kb) and the mutated copy (*ispD::aphA3*, 1.4 kb) when using primers specific to the native locus (Fig. 2A). Transformation of the WT strain with the *ispD::aphA3* construct yielded kanamycin-resistant transformants. However, in addition to the 1.4 kb mutated copy, all recovered transformants additionally had the WT copy of *ispD* by colony PCR; thus, these transformants were merodiploid for *ispD* (Fig. 2A, lane 1). To generate a clean *ispD* mutant, *ispD* was first inserted at a distinct location in the *Nm* genome under the control of an IPTG-inducible *lac* promoter; the native *ispD* was then deleted in the presence of IPTG. The *P_lac_::ispD* complemented mutants were confirmed by PCR to only contain the *ispD::aphA3* at the native locus (Fig. 2A, lane 2). Using this strategy, *ispD* mutants complemented with either *ispD*_CNM3_ (the *Nm*UC allele) or *ispD*_MC58_ (the *Nm* allele)—CNM3/*P_lac_::ispD*_CNM3_/Δ*ispD* (CC) and MC58/*P_lac_::ispD*_MC58_/Δ*ispD* (MM)—were created. When plated in the presence of 1,000 µM IPTG, both complemented mutants displayed robust growth; when plated without IPTG, both mutants displayed minimal growth due to the leaky control of the *lac* promoter, and MM appeared to grow slightly better than CC (Fig. 2B). These data indicated that *ispD* was also essential for *Nm*.

To test whether *Nm* or *Nm*UC aerobic growth was influenced by the distinct IspD variants, two additional complemented mutants were generated: CNM3/*P_lac_::ispD*_MC58_/Δ*ispD* (CM) and MC58/*P_lac_::ispD*_CNM3_/Δ*ispD* (MC). Subsequently, these four complemented mutants (CC, MM, CM, and MC) were plated with increasing concentrations of IPTG. The mutants with the *ispD*_MC58_ *Nm* allele (CM and MM) grew robustly aerobically at 10 µM IPTG, while the two mutants with the *ispD*_CNM3_ *Nm*UC (*Ng*-like) allele (CC and MC) required at least 100 µM IPTG to reach similar growth (Fig. 2C). Thus, regardless of the strain background, IspD_MC58_ promoted robust aerobic growth at lower IPTG inductions, that is, lower *ispD* levels. The induction of *ispD* transcription was at similar levels for the complemented mutants at the same IPTG concentrations. qRT-PCR analyses showed that *ispD* expression of CM grown on 10 µM IPTG is 118 ± 58% of CC grown on 10 µM IPTG (*P* = 0.7865). These results suggested the IspD_MC58_ variant might be more efficient in promoting aerobic growth.

### The gene conversion altered *ispD* DNA sequence and IspD protein in *Nm*UC

**Fig 3:**
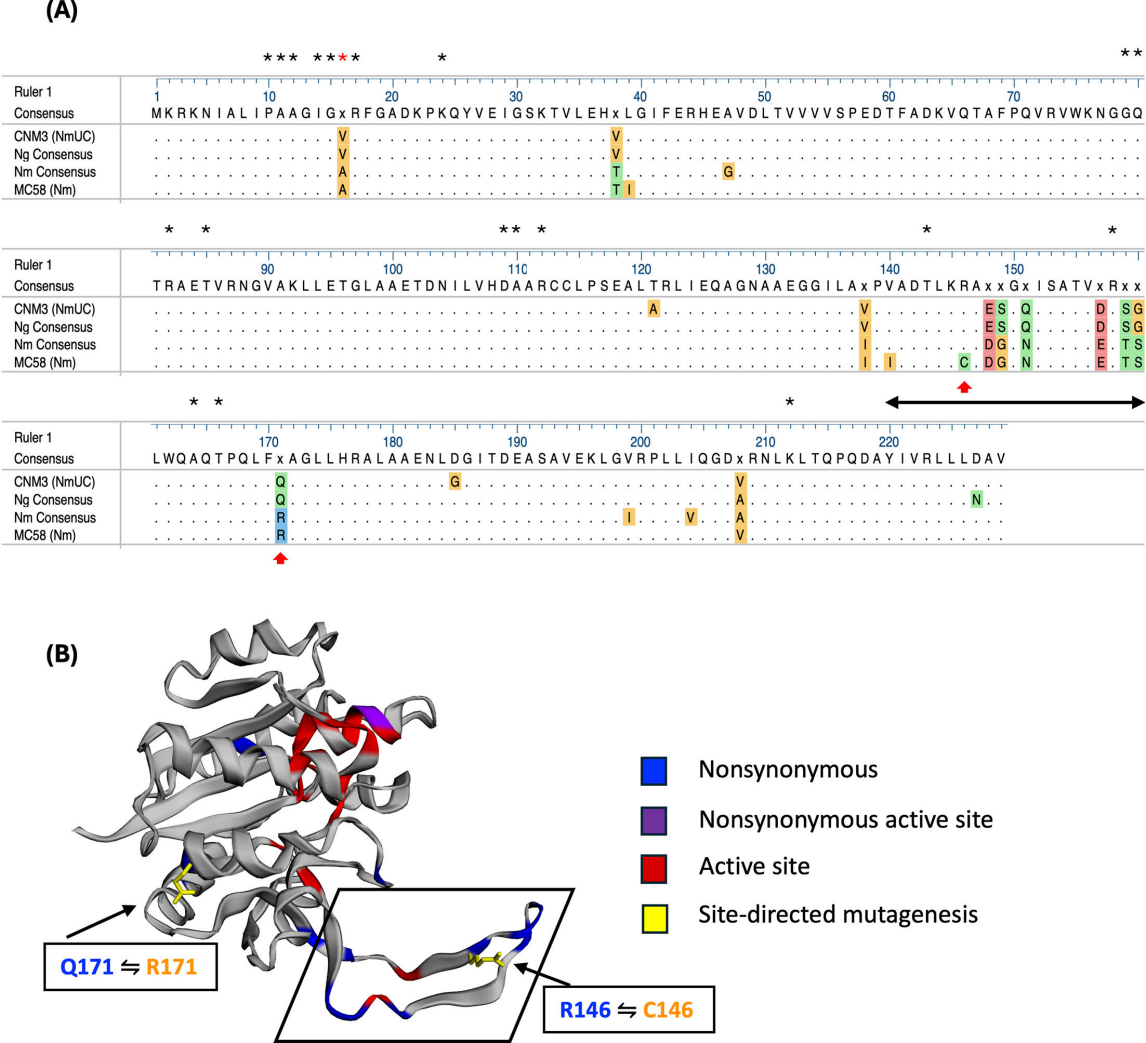
(**A**) Amino acid sequence comparison between *Nm*UC, *Ng*, and non-clade *Nm* IspD proteins. Consensus protein sequences for *Ng* and *Nm* were determined by aligning the 10 most common variants from each species in the PubMLST database (25). Amino acids that are different from the consensus of this multi-alignment are colored based on their side chain chemistry: orange, nonpolar; green, polar (neutral); red, polar (negatively charged); and blue, polar (positively charged). Asterisks mark active site residues. The active site residue that is nonsynonymous between the CNM3 and MC58 (residue #16) is marked with a red asterisk. The hook subdomain residues (#140–160) are denoted with a black double-sided arrow. Red arrows mark residues altered by site-directed mutagenesis. (**B**) 3D model of meningococcal IspD monomer. Residues are colored based on feature: blue, nonsynonymous residues between MC58 and CNM3; and red, active site residues; purple, nonsynonymous active site residue. Nonsynonymous residues 146 (also a subdomain residue) and 171 have their side chains displayed in yellow. Residues were mutated to match the corresponding residue on the other IspD type (CNM3 **⇋** MC58). The structure was generated with EzMol (26).

The recombination event replaced 85% of the meningococcal *ispD* coding sequence (588 out of 691 nucleotides) at the 5′ end with gonococcal sequence (8). The *ispD* allele 302 (*ispD*_302) of CNM3 was a characteristic of the clade (11); 259 out of 261 *Nm*UC isolates (99.2%) have been identified to contain this *Ng*-like allele. Alignment of the amino acid sequences showed that IspD_CNM3_ (*Ng-*like) and IspD_MC58_ (*Nm*) proteins differ by 15 residues (Fig. 3A). In addition, IspD consensus sequences for *Nm* and *Ng* were compiled from the 10 most common alleles in the PubMLST database. The *Nm* consensus had 16 residue differences from the *Ng*-like *ispD*_302 of *Nm*UC, of which 10 of these different residues were shared with *Nm* MC58 (Fig. 3A). The *Ng* IspD consensus sequence differed from *ispD*_302 by four residues at the 3′ (*Nm* retained) end of the *Nm*UC sequence.

The crystal structure of the *E. coli* IspD protein has been reported (27). Twenty active-site residues have been identified, as well as a hook subdomain that interlocks with a corresponding hook on another IspD protein to mediate dimer formation (Fig. S2). Our modeling of IspD_CNM3_ and IspD_MC58_ protein sequences onto the *E. coli* IspD structure showed that they were structurally identical. Aligning the CNM3 and MC58 IspD protein sequences to that of *E. coli* IspD revealed their active site and subdomain residues (Fig. 3B). Of the 15 nonsynonymous IspD_CNM3_ residues that differed from IspD_MC58_, V16A is a part of the active site and 8 other residues (V140I, R146C, E148D, S149G, Q151N, D157E, S159T, and G160S) are part of the hook subdomain.

The nonsynonymous changes between IspD_CNM3_ and IspD_MC58_ may contribute to the IspD-dependent aerobic growth phenotype (Fig. 2C). To investigate, one residue from the hook subdomain and one outside the hook subdomain were altered to the corresponding residue of the other variant (Fig. 3B). These residues were targeted due to different side-chain functionalities (R vs C at 146 and Q vs R at 171, Fig. 3A). The R_146_ residue of IspD_CNM3_ was mutated to C to match that of IspD_MC58_ and, conversely, the C_146_ of IspD_MC58_ was changed to R. Site-directed mutagenesis of residue 171 was performed analogously. In total, four single mutants and two double mutants were generated. CNM3 with IspD_CNM3_/R146C and CNM3 with IspD_MC58_/C146R were labeled CC_146_ and CM_146_, respectively; while those at residue 171 and double mutants were labeled correspondingly as CC_171_, CM_171_, CC_146/171_, and CM_146/171_. When plated with increasing concentrations of IPTG, none of these constructs showed an altered aerobic growth phenotype when compared to strains with corresponding WT IspD proteins (Fig. S3). The data suggested that residues 146 and 171 were not critical for the IspD variant-dependent aerobic growth phenotype observed in Fig. 2C. However, other changes in hook domain residues (V140I, E148D, S149G, Q151N, D157E, S159T, and G160S) suggest that this region represents an important difference between *Ng* and *Nm*.

### Aerobic growth curves of IspD variants

**Fig 4:**
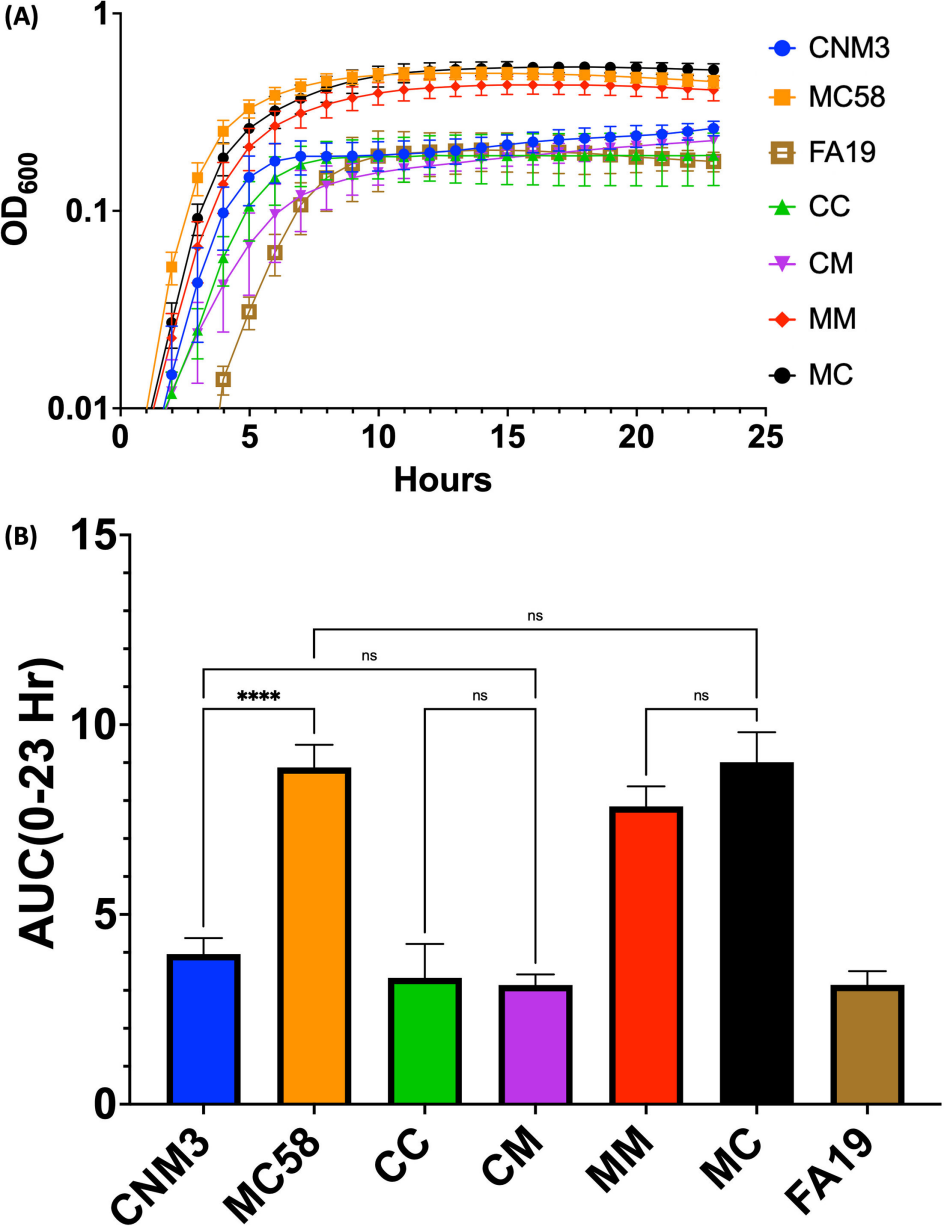
(**A**) Aerobic growth curves of *Nm*UC, *Nm*, *Ng*, and four *ispD*-complemented strains in GC broth. CC = CNM3::*ispD_Nm_*_UC_ Δ*ispD*; CM = CNM3::*ispD_Nm_* Δ*ispD*; MM = MC58::*ispD_Nm_* Δ*ispD*; and MC = MC58::*ispD_Nm_*_UC_ Δ*ispD*. Data shown are the means and standard deviations of three or more independent experiments for each strain. (**B**) Area under curve (AUC) analyses of 23 h aerobic growth of *Nm*UC, *Nm*, *Ng*, and four *ispD*-complemented strains. Data shown are the means and standard deviations of three or more independent experiments for each strain. Kruskal-Wallis test with Dunn’s multiple comparisons test was performed to compare all strains to CNM3: ****=*P* < 0.0001, ns = not significant.

Previous work (17) has indicated that broth cultures of *Nm*UC (CNM3) grow to a lower optical density than *Nm* (MC58) under aerobic conditions. To determine if IspD variants affected *Nm* and *Nm*UC growth, the WT strains and the complemented mutants were grown in GC broth supplemented with excess IPTG (1,000 µM) to ensure maximal induction. Growth curves showed that the strains behaved similarly based on *Nm*UC (CNM3, CC, and CM) or non-clade *Nm* (MC58, MM, and MC) backgrounds, regardless of the *ispD* allele (Fig. 4A). Strains with a CNM3 background grew to lower OD_600_ levels than those of MC58; the average maximum OD_600_ readings (AMO) for CNM3 and MC58 are 0.262 and 0.505, respectively. Complementing CNM3 with *ispD*_MC58_ (CM) did not enhance growth (AMO = 0.228), while complementing MC58 with the *ispD*_CNM3_ (MC) did not decrease growth (AMO = 0.540). The growth curves of the CNM3 background strains were similar to the *Ng* strain FA19 (AMO = 0.232) rather than *Nm*. Area under curve (AUC) analyses of the growth curves showed that AUCs of CNM3 strains were approximately a third to a half of the AUCs of MC58 strains, regardless of *ispD* allele (Fig. 4B). These data confirmed aerobic growth differences between *Nm*UC and *Nm* backgrounds. However, while IspD_MC58_ promoted better aerobic plate growth than IspD_CNM3_ at low levels of IPTG induction (1 and 10 µM IPTG, Fig. 2C), the IspD variant did not affect aerobic growth when IspD expression was maximally induced (Fig. 2C and 4).

### Increased *ispD* expression enhanced anaerobic survival of *Nm*

**Fig 5:**
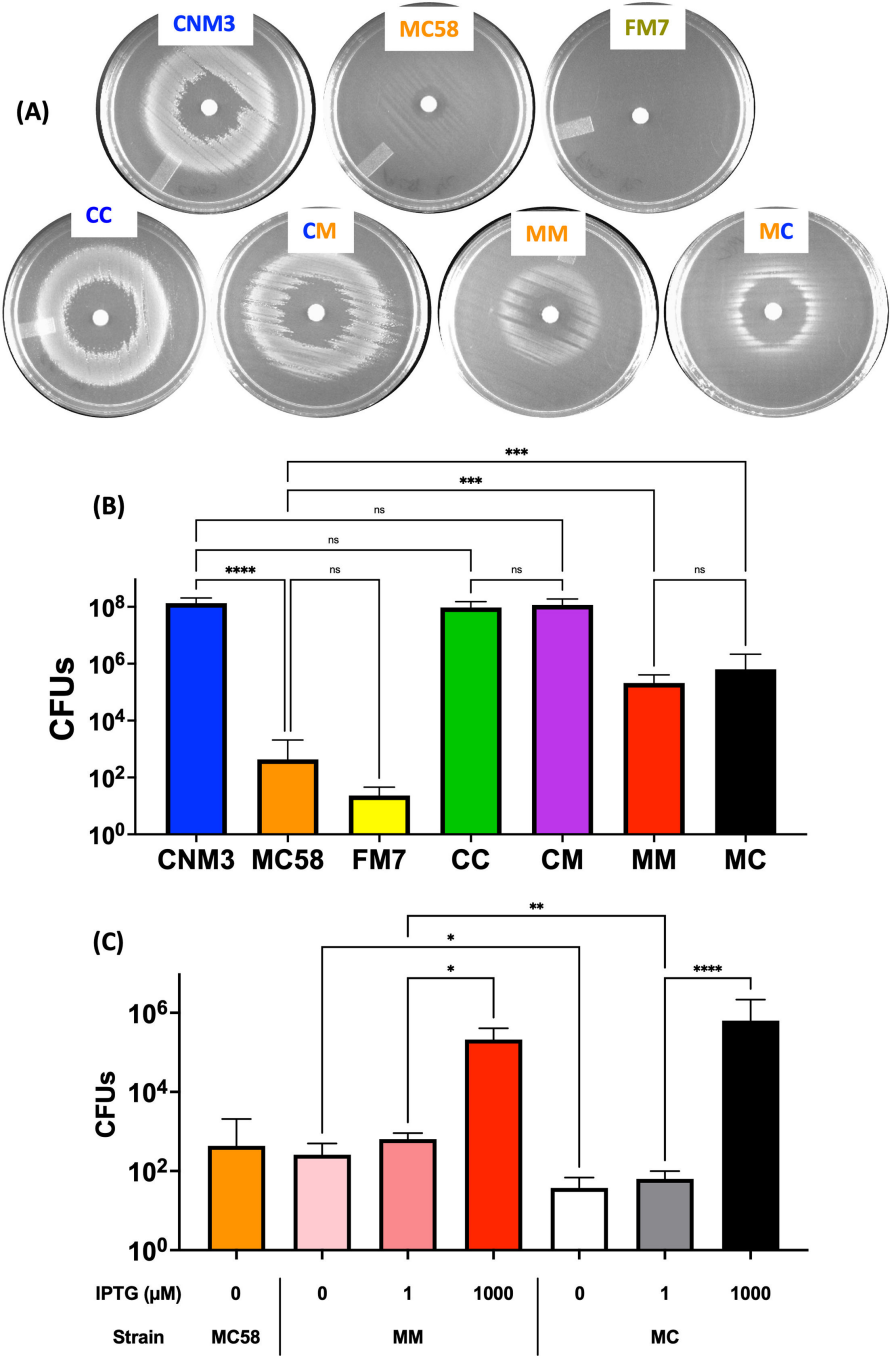
Survival of *Nm*UC, *Nm*, and *ispD*-complemented strains under anaerobic conditions. CC = CNM3::*ispD_Nm_*_UC_ Δ*ispD*; CM = CNM3::*ispD_Nm_* Δ*ispD*; MM = MC58::*ispD_Nm_* Δ*ispD*; and MC = MC58::*ispD_Nm_*_UC_ Δ*ispD*. Plates were incubated in anaerobic jar with <0.1% oxygen (9, 28). (**A**) Anaerobic survival of *Nm*UC, *Nm*, and four *ispD*-complemented strains on GC plates supplemented with 1,000 µM IPTG and sodium nitrite on discs. FM7 with a frameshifted *aniA* and a premature stop codon in *norB* serves as a negative control. the plates shown are representative of the anaerobic survival experiments repeated seven or more times. (**B**) Quantitation of anaerobic survival on GC plates. The mean values and standard deviations of colony counts (*n* ≥ 7 experiments per strain) are shown. Kruskal-Wallis test with Dunn’s multiple comparisons test was performed to compare strains: ***=*P* < 0.001, ****=*P* < 0.0001, ns = not significant. (**C**) Anaerobic survival of complemented *ispD* mutants with the MC58 background on GC plates supplemented with sodium nitrite and varied concentrations of IPTG. The mean values and standard deviations of colony counts are shown (*n* ≥ 4 experiments per strain). Kruskal-Wallis test with Dunn’s multiple comparisons test was performed to compare strains: *=*P* < 0.05, **=*P* < 0.01, ****=*P* < 0.0001.

While the non-clade *Nm* MC58 displayed greater aerobic growth over the *Nm*UC CNM3 (Fig. 4), previous work (9, 17) has shown that *Nm*UC has an improved ability to survive under microaerobic/anaerobic conditions in comparison to MC58. This is facilitated at least in part by a separate gene conversion–acquisition of the gonococcal AniA-NorB denitrification apparatus (9, 17). The role of IspD in anaerobic survival (an environment of <0.1% oxygen) (28) was tested using WT and *ispD*-complemented mutants in the presence of 1,000 µM IPTG (Fig. 5A). Non-clade *Nm* strain FM7 with frame-shifted *aniA* and *norB* was included as a negative control and no visible growth was seen on the plate. The strain background had the largest effect on post-anaerobic survival. In both CC and CM, anaerobic survival was comparable to the WT CNM3; all three strains had the gonococcal AniA-NorB denitrification apparatus. Of note, the IPTG-inducible complemented mutants, MM and MC, had similar survival to each other but both had significantly better anaerobic survival than the parental WT strain MC58, even though these strains contain the same meningococcal *aniA* and *norB*.

To quantify anaerobic survival, bacteria were recovered from plates and the viable CFUs were determined by plating. As shown in Fig. 5B, strains with an *Nm*UC background (CNM3, CC, and CM) had high viable CFUs (~10^8^). WT MC58 had few viable bacteria (<10^3^), as did FM7. The two complemented mutants of *Nm* background, MM and MC, had significantly higher CFUs (~10^5^) relative to that recovered from WT MC58 (*P* < 0.001). While WT MC58 and complemented strains carry a single non-clade *ispD*_MC58_ allele, WT MC58 has *ispD* under the control of the native promoter, whereas *ispD* is under the control of a *lac* promoter in MM and MC. Thus, the CFU differences may be due to higher IPTG-induced *ispD* expression in MM and MC versus that derived from the native promoter. The low *ispD* transcription, detectable by qRT-PCR, in the absence of the IPTG inducer (~15% of WT) confirmed the “leaky” *P_lac_*-controlled expression of *ispD* (Fig. S4); this leaky expression resulted in minimal viable counts recovered under no IPTG induction (Fig. 5C). Viable counts increased by 3–4 logs when grown in the presence of 1,000 µM IPTG relative to the samples with 1 µM IPTG (MM: *P* < 0.05, MC: *P* < 0.0001). These data demonstrated that higher *ispD* expression enhanced anaerobic survival of MM and MC strains with an *Nm* background. Interestingly, MM had significantly higher CFUs compared to MC at a low induction with 1 µM IPTG (*P* < 0.01), but the difference between variants was negligible at robust expression of *ispD* (1,000 µM IPTG). This profile was consistent with the aerobic growth phenotypes seen on plates (1 vs 100 µM IPTG, Fig. 2C) and supported the idea that IspD_MC58_ might have a higher enzymatic activity that also promotes anaerobic survival on plates when the amount of IspD was limited.

### The gene conversion event altered *ispD* expression in *Nm*UC

**Fig 6:**
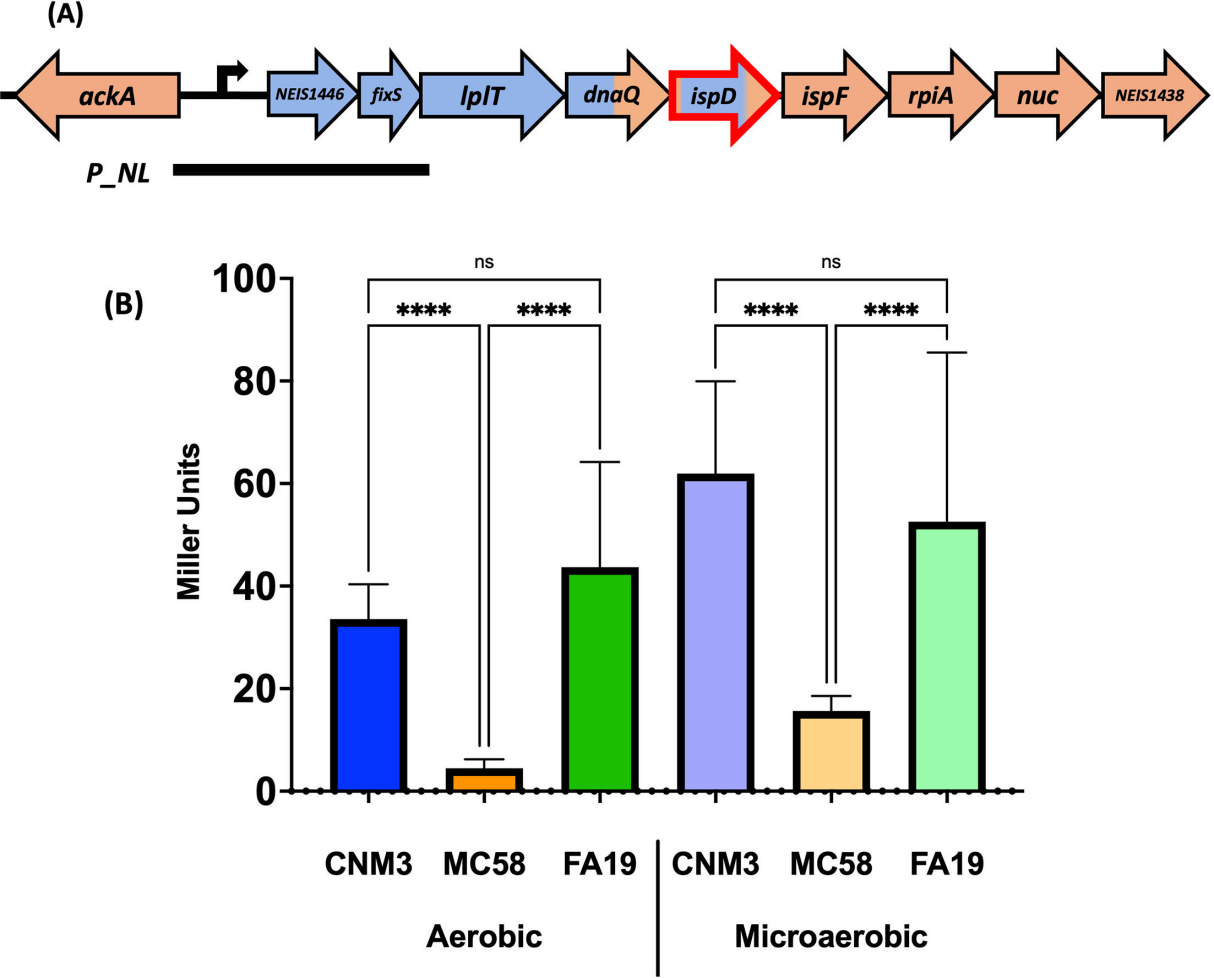
Expression of *NEIS1446-ispD*. (**A**) Schematic of the operon promoter sequence of *Nm*UC cloned into the *P_NL* translational reporter. Meningococcal sequence is colored in orange; gonococcal sequence is colored in blue. (**B**) β-Galactosidase activities of *P_NL* reporters in *Nm*UC CNM3. The origins of reporter sequences (*Nm*UC-CNM3, *Nm-*MC58, or *Ng-*FA19) and growth condition (aerobic or microaerobic) are labeled on the *x*-axis. Data shown are means and standard deviations of three or more independent experiments for each strain. Ordinary one-way ANOVA with Šídák’s multiple comparisons test was performed to compare the *P_NL* reporters: ****=*P* < 0.0001, ns = not significant.

The data suggested that *ispD* expression influenced microaerobic and anaerobic survival. Thus, we investigated whether the gene conversion event altered the expression of the *ispD* operon in *Nm*UC. The SNP density plots (Fig. 1B) demonstrated that the recombination event had replaced the nucleotide sequence upstream of *ispD*, including the potential promoter in the intergenic region upstream of *NEIS1446*. Translational reporters were generated from the region covering the exchanged intergenic sequence from the divergently transcribed *ackA* to the 5′ end of *lplT* (*P_NL*) (Fig. 6A). Reporters of the promoter sequences from CNM3 (representative of *Nm*UC), MC58 (non-clade *Nm*), and FA19 (*Ng*) were integrated into permissive *proAB* loci on the CNM3 genome as a single copy via homologous recombination (29) and the promoter activities of aerobic and microaerobic cultures were assessed by β-galactosidase assay (Fig. 6B). To achieve a microaerobic environment (~6–12% O_2_, normal atmosphere is 21%) (28), a 12 mL broth culture supplemented with 5 mM nitrite was incubated in a sealed 15 mL tube and shaken at 100 rpm. This is a previously established microaerobic growth condition (17) that has been confirmed to induce *fnr*, which encodes the major regulator for adaptation to oxygen-limiting conditions. The CNM3 reporter had activities comparable to those of the *Ng* reporter, and both reporters had significantly higher activities than those of the MC58 reporter under both aerobic and microaerobic conditions (*P* < 0.0001). These data indicated that the integration of gonococcal DNA into the *Nm*UC altered the expression of the operon and, by extension, the expression of *ispD* to levels significantly higher than that originated from the non-clade *Nm* sequence.

## DISCUSSION

Over the last decade, *Nm*UC has emerged as a cause of clusters and outbreaks of male urethritis (7–9, 11). Part of *Nm*UC’s evolution is the integration and conservation of gonococcal DNA at several locations in the *Nm* genome, including an operon containing *ispD* (8, 19). This operon is a genomic characteristic of the clade and is present in the vast majority of *Nm*UC isolates (245 out of 261 or 93.9%). The *Ng*-like *ispD* in the operon is present in nearly all *Nm*UC isolates (259 out of 261 or 99.2%) and all of 52 most recent isolates from 2016 to 2022 (11). While the operon encodes proteins with diverse biological activities, we focused on how the gonococcal DNA conversion event influenced *ispD* expression and *Nm*UC growth. In this study, we showed that the gonococcal *NEIS1446-ispD* gene conversion contributes to the pathobiology of *Nm*UC. We demonstrated that the *NEIS1446-NEIS1442* (*ispD*) sequence change increased expression of the operon. While other genes in the operon may also contribute to urogenital pathogenesis and remain to be characterized, the data presented show that IspD is specifically contributing to enhanced microaerobic and anaerobic survival as a result of the conversion event to an *Ng* homolog. This is the first report revealing a link between the cellular levels of precursors/electron carriers and anaerobic survival in *Neisseria* as well as providing additional data on how gene conversion events facilitate new niche adaptation.

Based on homology to IspD in *E. coli*, IspD is predicted to be a part of the MEP pathway and thus involved in the synthesis of isoprenoid (terpenoid) precursors (21, 22) (Fig. 1). Isoprenoids are a diverse class of metabolites that serve as precursors for cellular factors involved in essential pathways such as the electron transfer chain (30, 31). For example, nitrate and nitrite reductases, important in microaerobic growth and survival, generate a proton gradient that utilizes isoprenoid quinones and ubiquinol as electron carriers (32). In *E. coli*, the nitrate reductase expressed under anaerobic and microaerobic conditions catalyzes the reduction of nitrate to nitrite, coupled with the oxidation of menaquinol to generate a proton gradient. In *Nm* and *Ng*, the ubiquinol pool transfers electrons directly to NorB to reduce nitric oxide, supporting oxygen-independent respiration (33, 34). IspD has been shown to be essential in other gram-negative bacteria including *Salmonella enterica* (24) and *E. coli* (23), and we demonstrated that *ispD* is also essential in *Nm*.

The operon containing *ispD* was previously shown (19) to contain at least seven genes (*lplT-NEIS1438*) in *Ng*. In *Ng*, the downstream *nuc* (Fig. 1) encodes a nuclease involved in biofilm formation and remodeling (19). Our linkage analysis found that *ispD* was co-transcribed with the eight flanking genes in *Nm*UC (Fig. S1). Under the control of an IPTG-inducible promoter, strains with the meningococcal *ispD*, *ispD*_MC58_, had enhanced aerobic growth, whereas strains with the gonococcal-like *Nm*UC *ispD*, *ispD*_CNM3_, required higher *ispD* expression to achieve similar growth, regardless of the strain background (Fig. 2C). The recombination event in *Nm*UC resulted in a gonococcal-like IspD protein that differs from the *Nm* IspD_MC58_ by 15 amino acids (Fig. 3A). Many of the amino acid differences are in the hook domain, suggesting these alterations may affect dimerization of IspD proteins and contribute to activity differences between *Ng* and *Nm* IspDs.

The meningococcus has been proposed to be on an evolutionary trajectory as an aerobe in the oxygen-rich nasopharyngeal niche (35). In contrast, *Nm*UC strains (e.g., CNM3) have been shown to survive robustly, while *Nm* (e.g., MC58) negligibly, after anaerobic incubation (9). We found that the IspD variant did not affect the anaerobic survival phenotype in the *Nm*UC background, either visually or by CFU count (CC and CM, Fig. 5). However, while there was no difference between MM and MC post-anaerobic incubation, both complemented MC58 mutants survived significantly better than the parental WT MC58, indicating that the increased anaerobic survival is a result of complementing with *P_lac_*-controlled expression of *ispD*. Varying the amount of inducer supported this idea: higher IPTG concentrations increased CFUs of MM and MC strains (Fig. 5C), indicating that higher *ispD* expression enhanced anaerobic survival. This hypothesis is corroborated by the translational reporter data (Fig. 6). Reporters of the *Ng* FA19 and *Nm*UC CNM3 sequences showed significantly higher activities than that of the *Nm* MC58 reporter under both aerobic and microaerobic conditions, indicating increased expression of the operon genes in *Nm*UC, including *ispD* and *ispF*. This increased expression of *ispD* may result in increased production of ubiquinone, and thus more efficient electron transfer to terminal electron acceptors during respiration (33, 34). Taken together, this suggests that the recombination of gonococcal sequence into the *Nm*UC genome yields higher expression of *ispD* in oxygen-limiting conditions and, together with the gonococcal AniA/NorB denitrification apparatus, enhances anaerobic survival relative to non-clade *Nm*. While expression of other genes in the operon has also increased, their effect(s) on *Nm*UC is unknown and remains a point of interest. Of note, *fixS* is a *cbb_3_*-type cytochrome oxidase assembly protein, and the gonococcal *cbb_3_*-type cytochrome has been implicated in the respiration of nitrite during anaerobic growth (33, 34).

*Nm*UC has emerged as a urogenital pathogen causing male urethritis (9). The human urethra is a microaerobic/anaerobic environment where oxygen is limited (16, 36–38): 0.47–51.5 mmHg O_2_ (0.06–6.94% O_2_) in the urinary tract. In addition, the female vagina is also a microaerobic environment with 15–35 mmHg O_2_ (2.02–4.71%) (16). We have previously shown that another gonococcal gene conversion in *Nm*UC, the *Ng* denitrification apparatus, AniA and NorB, contributes to enhanced anaerobic survival of *Nm*UC (17). This is corroborated by the data in this study, in which *Nm*UC strains (e.g., CNM3 and derivatives) displayed enhanced survival under anaerobic conditions. In contrast, the complemented MC58 mutants, which both have the less anaerobically efficient meningococcal AniA-NorB denitrification apparatus, survive significantly better under anaerobic conditions when *ispD* expression was increased. Thus, our data support that the replacement of *NEIS1446-ispD* with the gonococcal homolog in *Nm*UC increased *ispD* expression and contributed to *Nm*UC microaerobic and anaerobic survival.

## MATERIALS AND METHODS

### Bacterial isolates and growth conditions

Bacterial strains used in this study are listed in Table S1 of supplemental material. CNM3 served as the representative of *Nm*UC (9); MC58 served as the representative of non-clade *Nm*; *Neisseria* were cultured as described in Tzeng et al. (17). Strains were grown on GC base agar supplemented with 0.4% glucose and 0.68  mM Fe(NO_3_)_3_ at 37°C and 5% CO_2_, or in GC broth with the same supplements and temperature and 0.043% NaHCO_3_ as the CO_2_ source. Brain heart infusion (BHI) media with 1.25% fetal bovine serum was used with kanamycin selection. The following antibiotic concentrations (μg/mL) were used for *Neisseria*: kanamycin, 80; chloramphenicol, 5; and erythromycin, 3. The following antibiotic concentrations (μg/mL) were used for *E. coli*: kanamycin, 50; and ampicillin, 100. A 3 mL culture in a 50 mL conical tube at 200  rpm was defined as the aerobic growth condition. A 12 mL broth culture with 5 mM nitrite in a sealed 15 mL tube shaking at 100  rpm was defined as the microaerobic growth condition, which has been confirmed by qRT-PCR measurement of *fnr* expression being induced under oxygen limitation (17). Complemented mutants were grown in the presence of 1,000 µM IPTG, unless otherwise stated.

### PCR linkage analysis and quantitative RT-PCR

RNA extraction and cDNA synthesis were performed as described in Tzeng et al. (17). Cultures collected at mid-log phase or after overnight microaerobic growth were treated with RNAprotect (Qiagen). Total RNA was isolated using RNeasy minikit (Qiagen), treated with Turbo DNase (Invitrogen), and purified with Quick RNA microprep kit (Zymo). The cDNA samples were obtained by reverse transcription of total RNA (0.5 µg) using iScript Reverse Transcription Supermix (Bio-Rad). To measure the transcription of *ispD*, qRT-PCR was performed using the SYBR green detection method (Bio-Rad). The internal control for normalization was 16 s rRNA. Each reaction was performed in duplicate using primers ispD qF3 and ispD qR3.

For the PCR linkage analysis of the operon, cDNA samples were obtained by reverse transcription of total RNA (0.5 µg) using the Maxima H Minus First Strand cDNA Synthesis Kit (Thermo Fisher) with CTS281 or ispF-qR1 as the primer. PCR was performed on the cDNA samples with the primer combinations listed in Fig. 2B using Taq polymerase (Roche) and Taq Extender (Agilent).

### Construction of *ispD* deletion and complemented mutants

All primers used for the genetic manipulation are noted in Table S2 in the supplemental material. A fragment upstream of *ispD* (5n) was obtained by PCR using primers ispD-5F1 and ispD-5RA3 and sequence downstream of *ispD* (3a) was amplified with ispD-3FA and ispF3R with Q5 polymerase (New England Biolabs) and CNM3 genomic DNA as the template. The *aphA3* cassette fragment (A3) was obtained using aphA3-SmF and aphA3-SmR. Mixtures of A3 and 3a were used as the template for the first round of overlapping PCR with primers aphA3-SmF and ispF3R. The resulting fragment was mixed with 5a and used for the second PCR with primers ispD-5F1 and ispF3R to obtain the final construct (5n-A3-3a), in which ~600 of the 690 bp *ispD* coding sequence has been deleted and replaced with the *aphA3* cassette.

To generate the complemented mutants, *ispD* was amplified from genomic DNA using ispD-PacI and ispD-PmeI. The resulting fragment, along with plasmid pGCC4 (a gift from Hank Seifert) (Addgene plasmid no. 37058), was digested with PacI and PmeI restriction enzymes. The fragment was ligated into the plasmid to generate the final construct with *ispD* under the control of a *lac* promoter and its native ribosomal binding site. The complement *ispD* was inserted into the genome, and the native *ispD* was subsequently deleted with the 5n-A3-3a construct in the complemented construct.

For the site-directed mutagenesis *ispD* strains, overlap extension PCR was used to generate the constructs. For example, one-half of *ispD* with the desired nucleotide mutation (R146C 3′) was obtained by PCR using primers CNM3_R146C_3F and ispD-PmeI, and the other half (R146C 5′) was amplified with ispD-PacI and CNM3_R146C_5R. The two overlapping fragments were mixed and underwent PCR with ispD-PacI and ispD-PmeI. The final construct was ligated into pGCC4 after digestion with PacI and PmeI. This process was repeated for each mutant.

### Assessing growth in broth culture and survival on plates

Strains inoculated into the culture at ~0.1 OD_600_ were grown under aerobic conditions. At mid-log phase (~0.5 of OD_600_), a cotton-tipped applicator was dipped into the culture and spread onto a quarter section of a GC plate supplemented with varying concentrations of IPTG. Plates were incubated overnight and imaged the next day using the Gel Doc XR (Bio-Rad).

For anaerobic plates, strains were grown to ~0.5 OD_600_ and spread with a cotton-tipped applicator over the entire plate two times in a crosshatch fashion. A 6 mm paper disc was set in the middle of the plate and 10 µL of 20% NaNO_2_ was spotted onto the disc to provide nitrite. The plates were incubated at 37°C for 24 h in an anaerobic jar (Oxoid) with the AnaeroPack anaerobic gas generator that generated an environment of <0.1% oxygen according to the manufacturer (Thermo Fisher) (28) and confirmed with an anaerobic indicator (VWR). The plates were removed from the jar and allowed to grow at room temperature for an additional 72 h to form sufficient density for visualization and colony counting. Plates were then washed with GC media and recovered cells were serially diluted for viable colony counts after plating on GC plates. As only survived viable bacteria can multiply and no enhanced aerobic growth of CNM3 relative to MC58 was found (Fig. 4A), the colony counts after aerobic growth reflect the differences in anaerobic survival. The assay was validated and controlled by the inability of *Nm* FM7 to survive on anaerobic plates because this strain has an inactive denitrification apparatus (39). FM7 was a control of anaerobic environment performed in parallel with all anaerobic survival assays.

For growth curves, strains were grown to 0.5 OD_600_ and then diluted to 0.005; 200 µL of this culture was added to a 96-well microtiter plate in triplicate. GC media were used as a blank. The plate was incubated in plate reader (BioTek Eon), shaking at 425 cpm for 9 min and then OD_600_ recorded every 10 min. AUC was quantified using Growthcurver R package (40).

### Reporter construction and β-galactosidase assay

Reporter construction and assays were performed as described in Tzeng et al. (17). Promoter fragments with flanking BamHI sites were obtained by PCR using primers neis1446-F-Bm and lplT-R-Bm with Q5 polymerase (New England Biolabs) and genomic DNA. The fragments were cloned into pLES94 as translational fusions (29). The length of the promoter insert and the orientation were confirmed with proL3 + lacZrev and neis1446-F-Bm + lacZrev, respectively. The resulting plasmids were confirmed by sequencing to have an in-frame fusion with *lacZ* lacking the ribosomal binding site and the ATG start codon (41), and then the plasmids were used to transform CNM3. Transformants positive for insertion into the designated *proAB* locus via homologous recombination were identified by colony PCR; the *proAB* genes involved in proline biosynthesis are not essential, thus are permissive for insertional disruption (29). β-Galactosidase activities were calculated using the formula: 1,000 × (OD_420_ − 1.75 × OD_550_)/(minute × mL × OD_600_) and represented as Miller units (42). Each reporter was assayed in triplicate.

### Analyses of *Neisseria* consensus IspD sequences

In the PubMLST database, 561 distinct *ispD* alleles were found in *Nm* and 74 *ispD* alleles were found in *Ng*. The 10 most common alleles for each species were identified using the Fields Breakdown tool (25), selecting for the loci *NEIS1442 (ispD*). These *ispD* alleles together represent 73.8% of all *Nm* and 98.3% of all *Ng* in PubMLST, respectively. The alleles were then translated and aligned using the Locus Explorer tool; the consensus sequence was recorded.

### Statistical analyses

Statistical analyses were performed using GraphPad Prism 10. Differences were considered significant when *P* < 0.05. The data of growth curve AUC and colony count were compared using Kruskal-Wallis test with Dunn’s multiple comparisons test because both data sets had more than two groups and the data did not satisfy parametric assumptions (the data were not normally distributed). The translational reporter data were analyzed using Ordinary one-way ANOVA with Šídák’s multiple comparisons test because the data set had more than two groups and the data satisfied parametric assumptions.

## SUPPLEMENTAL MATERIAL

Supplemental material iai.00350-24-s0001.docx Tables S1 and S2; Fig. S1 to S4.

ASM does not own the copyrights to Supplemental Material that may be linked to, or accessed through, an article. The authors have granted ASM a non-exclusive, world-wide license to publish the Supplemental Material files. Please contact the corresponding author directly for reuse.
