# Supplementary material for: Contribution of the gonococcal NEIS1446-ispD gene conversion to the pathobiology of the Neisseria meningitidis urethritis clade, NmUC
Source: Infect Immun. 2025 Feb 4;93(3):e00350-24. doi: 10.1128/iai.00350-24 (PMC11895467; doi:10.1128/iai.00350-24)
Supplement: Supplemental material — Tables S1 and S2; Fig. S1 to S4. [file iai.00350-24-s0001.docx]

| **Bacterial Strain** | **Description, Relevant Genotype and Phenotype** | **Reference or Source** |
| --- | --- | --- |
| CNM3 | *N. meningitidis*, *Nm*UC reference | (1) |
| MC58 | *N. meningitidis*, non-clade *Nm* reference | (1) |
| FA19 | *N. gonorrhoeae* reference | (1) |
| FM7 | Unencapsulated *N. meningitidis* FAM18 with *cssA::tetM* and frame-shifted *aniA* and *norB* | (1) |
| CC | CNM3 with ∆*ispD*::*aphA3*, complemented with CNM3 *ispD* under the control of *lac* promoter | This study |
| CM | CNM3 with ∆*ispD*::*aphA3*, complemented with MC58 *ispD* under the control of *lac* promoter | This study |
| MM | MC58 with ∆*ispD*::*aphA3*, complemented with MC58 *ispD* under the control of *lac* promoter | This study |
| MC | MC58 with ∆*ispD*::*aphA3*, complemented with CNM3 *ispD* under the control of *lac* promoter | This study |
| CNM3 *P_NL* | CNM3 carrying the *P_NL::lacZ* reporter of CNM3 promoter | This study |
| MC58 *P_NL* | CNM3 carrying the *P_NL::lacZ* reporter of MC58 promoter | This study |
| FA19 *P_NL* | CNM3 carrying the *P_NL::lacZ* reporter of FA19 promoter | This study |
| CC_146_ | CNM3 with ∆*ispD*::*aphA3*, complemented with CNM3 *ispD* R146C under the control of *lac* promoter | This study |
| CC_171_ | CNM3 with ∆*ispD*::*aphA3*, complemented with CNM3 *ispD* Q171R under the control of *lac* promoter | This study |
| CC_146/171_ | CNM3 with ∆*ispD::aphA3*, complemented with CNM3 *ispD* R146C/ Q171R under the control of *lac* promoter | This study |
| CM_146_ | CNM3 with ∆*ispD::aphA3*, complemented with MC58 *ispD* C146R under the control of *lac* promoter | This study |
| CM_171_ | CNM3 with ∆*ispD::aphA3*, complemented with MC58 *ispD* R171Q under the control of *lac* promoter | This study |
| CM_146/171_ | CNM3 with ∆*ispD::aphA3*, complemented with MC58 *ispD* C146R/ R171Q under the control of *lac* promoter | This study |

**Table S1.** Strains used in this study.

**Table S2.** Primers used in this study.

| **Primer Name** | **Primer Sequence 5’** → **3’*** | **Reference or Source** |
| --- | --- | --- |
| ispD-5F1 | AATTCGATGTGGGCTTCCTCA | This study |
| ispD-5RA3 | ttcctcctagttagtcacccCATGCCGTTCAAAAATACCAAGT | This study |
| ispD-3FA3 | cctggagggaataatgacccCCGATTTGGGCATAGACATTTC | This study |
| ispF3R | ACCGCCAAACCCGATACTTC | This study |
| aphA3-SmF | GGGTGACTAACTAGGAGGAA | (2) |
| aphA3-SmR | GGGTCATTATTCCCTCCAGG | (2) |
| ispD-PacI | CCttaattaaAAACGGCGGGACGGACGCATGAA | This study |
| ispD-PmeI | AGCTTTgtttaaacTGAAGTTCAGACGGCATCGAGCAGC | This study |
| CNM3 R146C 3F | CGCTCAAGTGCGCAGAAAGCG | This study |
| CNM3 R146C 5R | CTTTCTGCGCACTTGAGCGTATCG | This study |
| MC58 C146R 3F | GCTCAAGCGCGCGGACGGTG | This study |
| MC58 C146R 5R | GTCCGCGCGCTTGAGCGTATC | This study |
| CNM3 Q171R 3F | CAGCTTTTTCGAGCGGGTTTGC | This study |
| CNM3 Q171R 5R | CAAACCCGCTCGAAAAAGCTGCG | This study |
| MC58 R171Q 3F | GCTTTTCCAAGCCGGGCTGC | This study |
| MC58 R171Q 5R | GCAGCCCGGCTTGGAAAAGCTG | This study |
| neis1446-F-Bm | GGCAGCggatccGGACTTCGCCGCTGCCGTTAT | This study |
| lplT-R-Bm | CCCAAggatCCTTTTTAGCGTCCATCGTTTTCC | This study |
| proL3 | GCATTGCTGCCGACTTCCTG | This study |
| lacZrev | ACGACGACAGTATCGGCCTCAGG | (2) |
| ispD-F-Eco | ccggaattcCCAAATTCGATGTGGGCTTCCTCA | This study |
| ispF-R-Eco | ccggaattcTTCGTCATCGTTTGTGTTCCTGA | This study |
| ispF-qR1 | GCGAGTTTGGGTTTCTGTG | This study |
| CTS281 | CGCCGTAAAGAAACATCCAGC | This study |
| nuc-qF2 | CGGAGATGAAACAGGCTTACG | This study |
| nuc-5F | ACGGTGCGGACGAAGTGAAC | This study |
| ispF-qF2 | CAGGGCTACGATGTCCACCA | This study |
| CTS286 | TTTGAAACTGACGCAGCCG | This study |
| ispD-5F1 | AATTCGATGTGGGCTTCCTCA | This study |
| lplT-3F | GCGGTATTGCTGTTTTTGTTTG | This study |
| fixS-3F | GCATTTTGATGGATGATGATTCG | This study |
| fixS-F-Bm | AAGGCGGatccGGAATTTATGCATTATTTCA | This study |
| ispD qF3 | AAAAATATCGCGCTGATTCC | This study |
| ispD qR3 | ATGCCGTCTGAACCTTATCG | This study |

* Nucleotides in red indicate changes made to introduce the site-directed mutation.

**
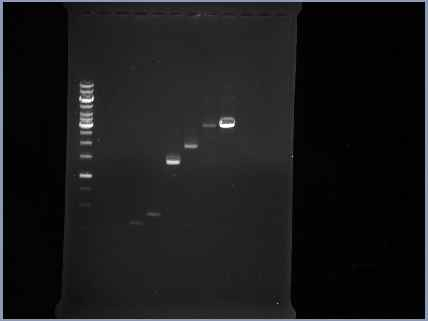

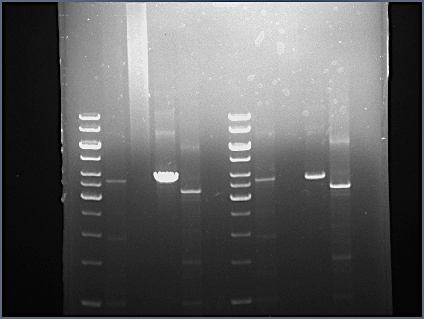

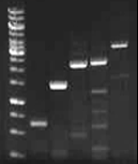
**

**CTS281**

**ispF-qR1**

+1

+6300

***NEIS1446***

***fixS***

***dnaQ***

**CTS281**

**ispF-qR1**

1 kb

3 kb

**(A)**

**(B)**

1

2

3

4

5

6

7

gDNA

**ispF-qR1**

**gDNA**

8

9

***nuc***

***rpiA***

***NEIS1438***

***ispF***

***ispD***

***lplT***

4

5

9

6

10

7

8

11

12

13

13

12

11

10

6 kb

9. nuc-qF2 + CTS281

10. nuc-5F + CTS281

11. ispF-qF2 + CTS281

12. CTS286 + CTS281

13. ispD-5F1 + CTS281

1. nuc-qF2 + ispF-qR1 5. ispD-5F1 + ispF-qR1

2. nuc-5F + ispF-qR1 6. lplT-3F + ispF-qR1

3. ispF-qF2 + ispF-qR1 7. fixS-3F + ispF-qR1

4. CTS286 + ispF-qR1 8. fixS-F-Bm + ispF-qR1

**Figure S1. Confirmation of the *NEIS1446 (HP)-NEIS1438 (abcT*) operon in *Nm*UC.** **(A) Map of overlapping PCR fragments**. The cDNA templates were made with reverse primers ispF-qR1 and CTS281. The number next to each bar notes their corresponding primer pairs and lane number in gel images in (B). **(B) Linkage PCRs.** Left and middle panels are PCR products using cDNA generated with the reverse primer ispF-qR1 and the right panel with reverse primer CTS281. The color of the bar next to the primer pair corresponds to the theoretical PCR product of the same color in (A). Primer pairs of 1-3 do not generate any PCR products because the forward primers are downstream of the reverse primers. The gDNA sample adjacent to lane 7 is the positive control for lane 7 of the left panel. The lane 2 in the middle panel is a RT(-) negative control for DNA contamination and the gDNA lane is a positive control for lane 8. White arrows mark the DNA size ladder at 6, 3, and 1-kb.

**Figure S2. The crystal structure of *E. coli* IspD dimer complexed with CDP-ME and Mg^2+^.** Residues corresponding to residues 146 and 171 of *Nm* IspD are noted with a red box. The location of the CDP-ME and Mg^2+^ substrates is marked with a blue box. The ribbon model is drawn from the file ID 1INI of the protein data bank (PDB) at [RCSB.org](http://www.rcsb.org/) (3, 4).

**Figure S3. IspD site-directed mutations do not affect the minimum inducer concentration required for robust growth.** Site-specific mutants were grown in broth cultures to mid-log phase (~0.5 OD_600_) and then spread onto GC plates. Data shown are representative of aerobic growth experiments repeated 3 times. CC mutants: CC146 = CNM3::*ispD_Nm_*_UC_ R146C; CC171 = CNM3::*ispD_Nm_*_UC_ Q171R; CC146/171 = CNM3::*ispD_Nm_*_UC_ R146C/Q171R. CM mutants: CM146 = CNM3::*ispD_Nm_* C146R; CM171 = CNM3::*ispD_Nm_* R171Q; CM146/171 = CNM3::*ispD_Nm_* C146R/R171Q.

**Figure S4. The complemented mutant with deleted native *ispD* has a low level of *ispD* transcription when uninduced, indicating a leaky promoter control.** Expression of *ispD* in aerobic cultures of CNM3 and CC without IPTG as determined by qRT-PCR (n = 3). Gene expression is normalized to that of CNM3 set as 100%. CC is a representative of *ispD* expression of all complemented mutants.

1. Tzeng YL, Bazan JA, Turner AN, Wang X, Retchless AC, Read TD, Toh E, Nelson DE, Del Rio C, Stephens DS. 2017. Emergence of a new Neisseria meningitidis clonal complex 11 lineage 11.2 clade as an effective urogenital pathogen. Proc Natl Acad Sci U S A 114:4237-4242.

2. Tzeng YL, Sannigrahi S, Berman Z, Bourne E, Edwards JL, Bazan JA, Turner AN, Moir JWB, Stephens DS. 2023. Acquisition of Gonococcal AniA-NorB Pathway by the Neisseria meningitidis Urethritis Clade Confers Denitrifying and Microaerobic Respiration Advantages for Urogenital Adaptation. Infect Immun 91:e0007923.

3. Richard SB, Bowman ME, Kwiatkowski W, Kang I, Chow C, Lillo AM, Cane DE, Noel JP. 2001. Structure of 4-diphosphocytidyl-2-C- methylerythritol synthetase involved in mevalonate- independent isoprenoid biosynthesis. Nat Struct Biol 8:641-8.

4. Berman HM, Westbrook J, Feng Z, Gilliland G, Bhat TN, Weissig H, Shindyalov IN, Bourne PE. 2000. The Protein Data Bank. Nucleic Acids Res 28:235-42.

**SUPPLEMENTAL MATERIAL BIBLIOGRAPHY AND REFERENCES CITED**
